# A Comparative Analysis of Local and Systemic Immunological Biomarkers in Females With Breast Implants and Capsular Contracture

**DOI:** 10.1093/asjof/ojae008

**Published:** 2024-02-21

**Authors:** Karlinde A Spit, Siham Azahaf, Christel J M de Blok, Yara Bachour, Kitty C M Castricum, Victor L J L Thijssen, Manon A H Oudejans, Thomas Rustemeyer, Prabath W B Nanayakkara

## Abstract

**Background:**

The etiology of capsular contracture (CC), the most common complication following breast augmentation, remains unclear. Chronic, fibrotic inflammation resulting in excessive fibrosis has been proposed as a potential mechanism.

**Objectives:**

In this study, we aimed to investigate the relation between biomarkers that are associated with inflammation and fibrosis and the severity of CC.

**Methods:**

Fifty healthy females were categorized into 3 groups: females with no-to-mild CC (Baker 1-2; *n* = 15), females with severe CC (Baker 3-4; *n* = 20), and a control group awaiting breast augmentation (*n* = 15). We assessed 5 biomarkers (galectin-1 [Gal-1], interferon-β [INF-β], interferon-γ [INF-γ], interleukin-6 [IL-6], and tumor necrosis factor-α [TNF-α]) in breast implant capsules and serum samples.

**Results:**

No significant differences in intracapsular cytokine levels were observed between the Baker 1-2 and the Baker 3-4 groups, as the levels were generally low and, in some cases, almost undetectable. In the blood samples, no significant differences in Gal-1, INF-γ, IL-6, or TNF-α levels were found within the 3 groups. We identified significantly increased levels of INF-β (*P* = .009) in the blood samples of females with severe CC, driven mainly by 3 extremely high values.

**Conclusions:**

The cytokines assessed in this study did not reflect the degree of CC among females with silicone breast implants. However, 3 females with severe CC, who all had prolonged silicone exposure, showed extremely elevated levels of INF-β in their serum samples. This possible association between prolonged silicone exposure and systemic inflammation in some females should be further investigated.

**Level of Evidence: 3:**

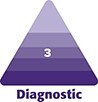

Since 1963, silicone breast implants have been used worldwide for breast reconstruction and augmentation. Although considered safe medical devices, up to 50% of females encounter complications related to implants within 15 years, with an estimated 10% of these complications occurring within 2 to 4 years postsurgery.^1-4^ The prevalence of these complications, both local and systemic, has sparked an ongoing debate about the safety of breast implants. General postoperative complications, including seroma, infection, and hematoma, are more frequently observed in reconstructive breast surgery compared with cosmetic breast surgery. The documented prevalence of these complications varies widely, ranging from 3% to 85%, 3% to 19%, and 0.2% to 9%, respectively.^5-7^

A particularly common, yet poorly understood, local complication is capsular contracture (CC), where the formation of a fibroblast-rich capsule around the implant leads to thickening and tightening, potentially leading to breast discomfort and distortion. This capsule consists mostly of fibroblasts that form collagen and some inflammatory cells, including macrophages, monocytes, and T cells.^8^ CC is estimated to occur in 2% to 15% of primary breast augmentation surgeries and 5% to 22% of secondary breast augmentation surgeries.^9-14^ The severity of CC is graded by the Baker classification, which ranges from 1 (normal capsule formation) to 4 (severely contracted capsule).^15^ The original Baker classification, introduced in 1978 and shown in Table 1, is still widely used in clinical practice. However, interobserver reliability and observer agreement of the Baker classification for CC are poor.^17^ Currently, there is no known biomarker associated with the severity of CC.

**Table 1.** The Original Baker Classification of Capsular Contracture

Class	Description
1	Breast absolutely natural; no one could tell breast was augmented
2	Minimal contracture; I can tell surgery was performed, but patient has no complaint
3	Moderate contracture; patient feels some firmness
4	Severe contracture; obvious just from observation

From Baker.^16^

Several risk factors contribute to the development of CC, including a smooth implant surface, subglandular placement, and reconstructive surgery. Surgical intervention remains the primary and most effective treatment. Previous techniques focused on capsulotomies, whereas current management is focused on capsulectomy and implant removal or replacement.^14^

Although the etiology of CC remains to be elucidated, hypotheses often center around the concept of low-grade chronic inflammation surrounding the implants. In addition to surgical strategies, there is growing interest in exploring pharmacological interventions targeting anti-inflammatory, antifibrotic, and antibacterial properties to prevent or treat CC effectively.

Authors of several studies have explored immunological factors that could potentially play a role in capsule contracture. In a study, Kyle and Bayat investigated fibroblasts from normal breast tissue and contracted and noncontracted breast implant capsules.^18^ In their findings, they indicated that fibroblasts within contracted capsules exhibited a cytokine profile that was both proinflammatory and profibrotic, contrasting with the characteristics of fibroblasts from normal breast tissue or noncontracted breast implant capsules. In their study, Tan et al showed an increased expression of tumor necrosis factor-alpha (TNF-α) in contracted breast implant capsules compared with noncontracted capsules.^19^ Even though TNF-α is primarily known for its proinflammatory role, its involvement in influencing fibrotic processes has also been documented.^20,21^ TNF-α and interleukin (IL)-6, important proinflammatory cytokines associated with several autoimmune diseases such as rheumatoid arthritis, are able to influence each other's secretion levels.^22^ Both TNF-α and IL-6 have become promising targets for therapeutic interventions in several inflammatory diseases.

Given that capsule inflammation and fibrosis involve T-cell immune responses, with Th1/Th17 T cells prevailing in contracted capsules, several other immunological biomarkers could theoretically contribute to CC. The signaling of Th1 effector cells is associated with M1 stimulation through the expression of interferon-γ (INF-γ).^23,24^ Galectine-1 (Gal-1), a prototype member of carbohydrate-binding proteins, has been persistently overexpressed in myofibroblasts and linked to keloid formation, tumorigenesis, and metastasis in various types of tumors, including breast tumor tissue.^25^ An elevated mRNA expression of Gal-1 may play a pivotal role in the development of myofibroblast-induced collagen secretion, thereby contributing to capsule thickening in CC.^26^ Lastly, interferon-β (IFN-β) has demonstrated antifibrotic and immunomodulatory properties, suppressing the activation and proliferation of fibroblasts and maintaining immune homeostasis through the promotion of regulatory T cells. An increased expression of IFN-β may reflect the prevention of Baker grading progression.^27^

An overview of these immunological biomarkers and their function is shown in Table 2.^28-30^

**Table 2.** Overview of the Immunological Biomarkers and Their Functions

Immunological biomarkers	Function
TNF-α	Strong proinflammatory cytokine that plays a pivotal role in many inflammatory diseases such as rheumatoid arthritis, psoriasis, and inflammatory bowel diseases. Modulation of TNF-α levels has become a very promising target of therapy for these diseases
Gal-1	Protein, overexpressed in myofibroblasts, which displays broad anti-inflammatory activities. Gal-1 is linked to keloid formation and collagen secretion
INF-γ	Type II interferon, produced by natural killer cells. Aberrant production has been associated with chronic autoimmune diseases such as inflammatory bowel disease
IFN-β	Type I interferon has demonstrated antifibrotic and immunomodulatory properties. Suppresses activation of fibroblasts
IL-6	Cytokine that has both pro- and anti-inflammatory activities. Levels are elevated in inflammatory autoimmune diseases such as rheumatoid arthritis

Gal-1, galectin-1; IL-6, interleukin-6; INF-β, interferon-β; INF-γ, interferon-γ; TNF-α, tumor necrosis factor-α.

In this exploratory study, we aim to investigate potential biomarkers associated with the severity of CC. We focus on 5 immunological biomarkers—Gal-1, IFN-β, IFN-γ, IL-6, and TNF-α—previously linked to inflammation-associated fibrotic processes.^18,19,26^ By examining their presence in breast capsules of females with varying CC grades and comparing local cytokine levels with serum measurements, we aim to investigate a potential correlation between local and systemic biomarkers. This approach aligns with the hypothesis that local immune reactions may trigger systemic responses, potentially explaining systemic symptoms that some females with breast implants report.

## METHODS

### Patients

Females were included between 2016 and 2019. Females who visited OLVG hospital location West, Amsterdam or Medical Centre Jan van Goyen, Amsterdam, in the Netherlands, for implant replacement or removal were eligible for inclusion. Females, who were on a waiting list for breast augmentation, were eligible for inclusion in the control group. Written informed consent was obtained from all participants. Ethical approval was granted by the local Medical Ethical Review Committee of VU University Medical Centre (reference number: 2015.052).

Females were included in Group 1 (Baker 1-2) if they presented with a clinically noncontracted capsule and underwent surgery for a replacement procedure or implant removal due to other reasons. Patients were included in Group 2 (Baker 3-4) if they presented with severe CC (preferably bilaterally) and underwent implant removal surgery with capsulectomy. Females on the waiting list for breast augmentation (thus without breast implants) were selected as a control group (Group 3). Participants were excluded if they were younger than 18 years, had relevant comorbidities, for example, chronic heart, lung, or liver diseases, had a history of cancer, had previous silicone injections, were current smokers or smokers in the past year, were pregnant, or used alcohol and/or drugs.

### Collection of Patient Material

Before surgery, each patient's medical history was collected along with baseline demographics. Baker grades were scored independently by 2 experienced surgeons. During the surgery, samples from the left and the right breast capsules were collected, and blood was drawn. All capsule samples were snap-frozen in liquid nitrogen directly after surgery and stored at a temperature of −80°C.

In the control group of females without implants, only blood was drawn. A total of 4 blood samples per patient were collected: 2 in ethylenediaminetetraacetic acid (EDTA) tubes and 2 in serum-separating tubes. One EDTA tube and one serum-separating tube were stored at −80°C; the other EDTA tube and serum tube were stored at −20°C. All samples were stored at the VU University Medical Centre, Amsterdam, until further analysis.

### Gene Expression Analysis

The expression levels of Gal1, IFN-β, IFN-γ, IL-6, and TNF-α mRNA in the breast capsules were evaluated using quantitative polymerase chain reaction (qPCR). From the snap-frozen capsules, 10 × 40 μm thin cryosections were collected, from which RNA was isolated using TRIzol Reagent (Invitrogen, Waltham, MA, USA) according to the supplier's protocol. The quality and concentration of the RNA were analyzed using NanoDrop ND-1000 (NanoDrop Technologies, Wilmington, DE, USA) and/or the Bioanalyser (Agilent Technologies, Santa Clara, CA, USA). Subsequently, 1 μg of RNA was reverse-transcribed into cDNA using the iScript cDNA Synthesis Kit (Bio-Rad, Hercules, CA, USA) according to the supplier's protocol. Then, milliQ was added to the cDNA up to a volume of 50 μL. For every primer combination, 1 μL of cDNA was used in a 25 μL PCR volume, containing 400 nM of the forward and reverse primers (Gal1 forward: TGCAACAGCAAGGACGGC, reverse: CACCTCTGCAACACTTCCA; INF-β forward: AAACTCATGAGCAGTCTGCA, reverse: AGGAGATCTTCAGTTTCGGAGG; INF-γ forward: CGAAAAGCTGACTAATTATTCGG, reverse: CTCTTCGACCTCGAAACAGC; IL-6 forward: GCCAGAGCTGTGCAGATGAG, reverse: CAGCTTCGTCAGCAGGCTG; TNF-α forward: GGCGTGGAGCTGAGAGAT, reverse: TGGTAGGAGACGGCGATG). PCR was performed on the CFX96 Real-Time PCR Detection System (Bio-Rad) using the SYBR Green mix (Bio-Rad) according to the manufacturer's protocol. CyclophilinA and β-actin were used as reference genes to normalize the expression of the target genes. Expression levels were calculated from the Ct values using the 2^(ΔCt) approach, with ΔCt = Ct target gene − average Ct reference genes. These converted values were used to compare the different patient groups.

### Serum Protein Level Analysis

To determine the Gal-1 and cytokine protein levels in the blood samples, a sandwich enzyme-linked immunosorbent assay (ELISA) was performed on patient-derived sera from all 3 groups. The ELISA was performed in maxisorb 96-well plates using specific ELISA kits, following the manufacturer's instructions: Gal-1 ELISA (R&D systems DY1152-05), IFN-β ELISA (R&D systems DY814-05), IFN-γ ELISA (Immunotools 31673539U1), IL-6 ELISA (Immunotools 31670069U1), and TNF-α ELISA (Immunotools 31673019U1). Absorbance was measured at 450 nm using a standard microplate reader, and the results are presented as the amount of protein per milliliter of serum.

### Statistical Analysis

Normally distributed values are presented as mean ± standard deviation (SD), whereas nonnormally distributed values are presented as median (interquartile range). In cases where values were not normally distributed, an Ln transformation was conducted. The comparison of qPCR outcomes between the 2 patient groups was conducted using an independent sample *t*-test. To compare the values derived from the ELISA between the control and the patient groups, analysis of variance tests were performed. In all tests, a threshold of *P* ≤ .05 was considered statistically significant. The test results were corrected for the variables of age, implant rupture, duration of implantation, and total number of breast surgeries. All tests were performed using SPSS software (IBM SPSS Statistics 26, Chicago, IL, USA).

## RESULTS

In total, 50 females were included in this study, of whom 15 were in Group 1 (Baker 1-2), 20 in Group 2 (Baker 3-4), and 15 in Group 3 (controls). A total of 68 breast implant capsules were collected since 2 females underwent unilateral surgery. The mean age of the participants was 42.5 years (SD 13.8), with implantation surgery conducted approximately 12.8 years ago on average (SD 1.8). Table 3 shows the baseline patient demographics per group.

**Table 3.** Baseline Characteristics Per Group

	Group 1, Baker 1-2 (*n* = 15)	Group 2, Baker 3-4 (*n* = 20)	Group 3, Controls (*n* = 15)	Mean	*P*-value
Age, mean (SD)	45.0 (6.2)	51.1 (13.7)	28.6 (7.4)	42.5 (13.8)	<.001
BMI, mean (SD)	23.0 (4.1)	24.0 (3.4)	22.6 (2.1)	23.3 (3.3)	.110
Duration of implantation, mean (SD)	10.7 (7.1)	14.5 (12.3)	—	12.9 (10.4)	.197
Number of breast operations, mean (SD)	1.4 (0.6)	1.9 (1.0)	—	1.7 (0.9)	.042
Ruptured implants, *n* (%)	2 (13)	1 (5)	—	3 (6)	.446

SD, standard deviation.

With regard to local gene expression, not all cytokine tests yielded detectable levels in the breast implant capsules. Gal-1 and TNF-α mRNA expressions were detectable in all 60 samples, whereas IL-6, INF-β, and INF-γ mRNA expressions were detectable in 58, 56, and 44 samples, respectively. No statistically significant differences were observed in any of the measured cytokines in the breast implant capsules between Groups 1 and 2 (Table 4).

**Table 4.** Median Local Cytokine mRNA Expression Levels (Interquartile Range) Per Group

	Group 1 (Baker 1-2)	Group 2 (Baker 3-4)	*P*-value	*P*-value after correction for confounders
Gal-1	1.009 (0.503-1.494)	0.949 (0.564-1.380)	.313	.851
INF-ß	0.172 (0.031-0.401)	0.096 (0.051-0.390)	.420	.833
INF-γ	0.001 (0.000-0.009)	0.001 (0.000-0.003)	.779	.939
IL-6	0.006 (0.002-0.0185)	0.007 (0.003-0.011)	.723	.640
TNF-α	0.191 (0.032-0.610)	0.234 (.078-0.440)	.996	.579

Expression levels are shown as 2^-deltaCt^ (see the Methods section for calculations). *P*-values were additionally corrected for age, number of breast operations, implant rupture, and duration of implantation. Gal-1, galectin-1; IL-6, interleukin-6; INF-β, interferon-β; INF-γ, interferon-γ; TNF-α, tumor necrosis factor-α.

**Figure 1.**
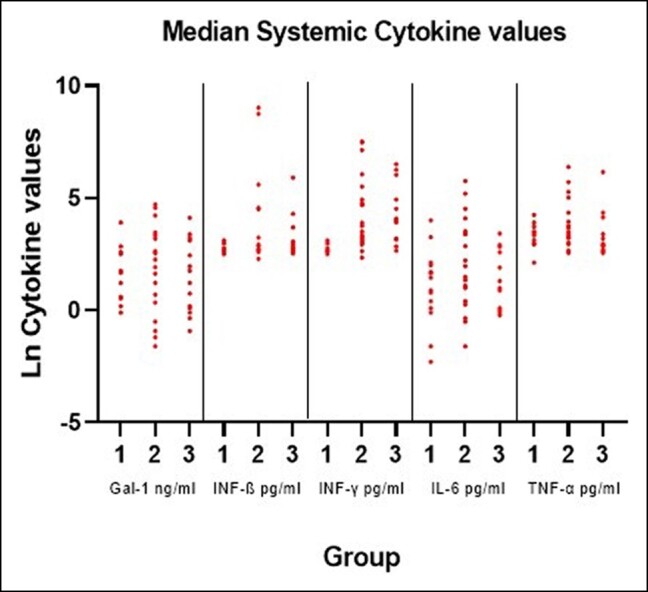
Systemic measured cytokines on logarithmic scale per cytokine and per group. Group 1: Baker 1-2; Group 2: Baker 3-4; Group 2: controls. Gal-1, galectin-1; IL-6, interleukin-6; INF-β, interferon-β; INF-γ, interferon-γ; TNF-α, tumor necrosis factor-α.

Although 4 out of the 5 cytokine levels were higher in Group 2 (Baker 3-4) compared with Group 1 (Baker 1-2) and Group 3 (control group), these differences did not reach statistical significance (Table 5). After adjustment for the variables of age, number of breast operations, implant rupture, and duration of implantation, INF-β remained significantly elevated in Group 2 when compared with the other 2 groups (*P* = .009), as illustrated in Figure 1. Notably, 3 females in Group 2 exhibited extremely high values of INF-β.

**Table 5.** Median Systemic Cytokines (Interquartile Range) Per Group

	Baker 1-2 (Group 1)	Baker 3-4 (Group 2)	Controls (Group 3)	*P*-value	*P*-value after correction for confounders
Gal-1	3.40 (1.70-12.30)	6.80 (0.60-28.30)	3.40 (1.10-22.50)	.885	.689
INF-ß	14.45 (13.60-15.30)	16.30 (14.30-91.30)	15.60 (14.30-21.20)	.111	.009
INF-γ	48.40 (12.30-90.10)	48.90 (23.70-245.10)	53.10 (23.40-139.00)	.400	.699
IL-6	2.40 (0.90-7.50)	4.40 (1.30-33.80)	3.70 (1.00-17.00)	.179	.390
TNF-α	28.50 (19.30-33.30)	28.90 (18.90-77.20)	19.20 (14.70-29.70)	.217	.671

Gal-1 is presented in ng/mL blood; INF-β, INFγ, IL-6, and TNF-α are in pg/mL blood. Analysis of variance was used to compare the 3 groups, additionally corrected for age, number of breast operations, implant rupture, and duration of implantation. Gal-1, galectin-1; IL-6, interleukin-6; INF-β, interferon-β; INF-γ, interferon-γ; TNF-α, tumor necrosis factor-α.

No correlations were observed between the locally and the systemically measured cytokine levels. Additionally, no differences in the systemically measured cytokine levels were found in Group 2 between females who presented with unilateral severe CC or bilateral CC.

Furthermore, a ruptured implant was discovered in 3 females during surgery (2 in Group 1 and 1 in Group 2), which was not associated with elevation of any of the local cytokine levels in the breast implant capsules. However, the sole female with an implant rupture from Group 2 exhibited extremely elevated systemic levels of all 5 cytokines in the blood samples, whereas the other 2 cases (from Group 1) did not.

## DISCUSSION

In this exploratory study, we aimed to identify a biomarker associated with the severity of CC. We explored biomarkers recognized for their relation to inflammation and excessive fibrosis, examining both local and systemic samples from females with breast implants and various grades of CC compared with a control group. The locally measured biomarker levels generally remained low, showing no discernible differences between females with severe or no-to-mild CC. We observed elevated levels of INF-β in the blood samples of females with severe CC compared with females with no or mild CC and controls. However, this was mainly driven by 3 extremely high values. Interestingly, these elevated systemic INF-β levels were not reflected by a similar increase in INF-β capsule levels. Lastly, no significant difference between the groups was found in the other biomarkers measured in the serum samples. Therefore, we did not identify a biomarker associated with the severity of CC, although the 3 observed females with severe CC and extremely elevated systemic INF-β levels are of interest for future research.

Unexpectedly and in contrast to the study by Tan et al, we did not observe an increase in TNF-α expression in severely contracted breast implant capsules compared with noncontracted capsules.^19,31^ It must be noted that the significant difference of 0.020 (*P* = .020) in TNF-α expression levels in this study was solely based on 2 samples.^19^ In their study, D’Andrea et al included 40 patients with CC and found a mean difference of 0.61 (*P* < .05) in TNF-α levels when compared with controls.^31^ However, the expression of target genes in this study was normalized with a single reference gene (Glyceraldehyde 3-phosphate dehydrogenase), diminishing the reliability of RT-PCR findings compared with our study, where multiple reference genes were used. Furthermore, TNF-α, IL-6, and INF-γ may not serve as suitable markers for Baker grading progression, given their nature as markers of acute inflammation.^8^ CC is more likely to represent the end result of a low-grade chronic inflammation process, potentially influenced by other internal and external factors.

Additionally, our results did not reveal a correlation between locally and systemically measured cytokine levels. Local cytokine levels generally appeared to be low. This is likely due to the fact that the initial inflammatory process and cytokine release may already be diminished by the time fibrosis occurs. However, considering the small part of the capsule that was used for analysis in this study, the potential for sampling errors cannot be entirely excluded. For future research, it is worth considering a focus on local measurements in the fluid surrounding the capsule, rather than within the capsule itself.

We noted a significant age difference among the 3 groups (*P* < .001), with the control group being significantly younger than females with CC. This is noteworthy because the concentration of major proinflammatory cytokines, such as IL-6 and TNF-α, typically correlates positively with age.^32,33^ However, even after adjusting for age, INF-β levels remained significantly elevated compared with the other groups.

The 3 females in this study, who demonstrated extremely high systemic INF-β levels, also exhibited elevated levels of other systemic biomarkers. These collective findings suggest a prevailing proinflammatory state. Notably, there were no reported relevant comorbidities or medication use that could account for the observed elevation in systemic biomarkers. Given their medical histories, the cause of this proinflammatory condition may be rooted in prolonged exposure to silicone. In particular, 2 females reported a cumulative implantation duration of 40 and 47 years, with respectively ruptured and intact implants. The third female underwent 5 breast surgeries caused by CC and implant ruptures. Correlations between implant duration and silicone leakage have been established in several studies.^34,35^ In particular, an association between the severity of the fibrotic response to a foreign body placement and an increasing number of breast operations has been observed in a previous study.^36^ However, it is worth noting that 2 other females from Group 1 (Baker 1-2) with ruptured implants showed no elevation in cytokine levels. This suggests that host immune responses to silicone (leakage) are likely multifactorial in nature. Genetic polymorphisms may explain the differences in the severity of foreign body reactions, as exemplified in a recent study on dermal fillers.^37^

This is the first study in which authors investigate several intracapsular biomarkers in relation to systemic biomarkers in females with CC, compared with a meticulously selected control group. The patients and groups were selected with care and strict inclusion and exclusion criteria. We believe this lays a robust foundation for further research into the immunological pathophysiology behind CC and the potential systemic immune responses triggered by breast implants.

Nevertheless, the exploratory nature of this study is circumscribed by the limited number of participants in each group. Although a statistically significant difference in INF-β levels among the 3 groups was observed, this outcome was influenced by extreme values from 3 patients in the severe CC group. Further research with a more extensive study population is imperative to ascertain whether INF-β genuinely plays a pivotal role in CC, potentially serving as a biomarker for its assessment. Interestingly, reports of systemic symptoms, such as fatigue and myalgia, from some females with breast implants underscore the importance of exploring the broader effects of INF-β. Its proinflammatory qualities suggest that, in susceptible individuals, INF-β could contribute to a systemic inflammatory response induced by silicone breast implants.

Lastly, the generally low levels of locally measured cytokines within breast implant capsules imply that capsule measurements may not be the optimal approach to measure the degree of CC. Our results suggest that future research should focus on systemic cytokine measurements. In addition, alternative measurement methods such as flow cytometry could be considered for future studies.

## CONCLUSION

In this explorative study, we provide a foundation for future research regarding immunological markers and CC. Despite our efforts, we did not identify a biomarker for CC, as the assessed cytokines in this study did not reflect the degree of CC among females with silicone breast implants. In addition, no correlation between capsule cytokine measurements and cytokine levels in serum samples was found.

However, we observed statistically significant elevated levels of INF-β in the serum samples of the group of females with severe CC. It is important to note that this observation was influenced by 3 extreme high values in this patient group. Interestingly, these 3 females exhibited prolonged silicone exposure, either due to a long implantation duration or due to ruptured implants. The potential systemic inflammatory response in susceptible females with severe CC and prolonged silicone exposure warrants further exploration in larger study populations.
